# On Opening the Joints

**Published:** 1861-01

**Authors:** E. S. Cooper

**Affiliations:** Professor of Anatomy and Surgery in the Medical Department of the University of the Pacific, San Francisco


*On Opening the Joints. On Opening freely the Large Joints for the purpose of Discharging Purulent Matter, as well as for the Better Treatment of Ulcerations of the Articular Surfaces. Remarks upon the Innocuousness of Atmosphere admitted into the Joints &c., &c.* By E. S. Cooper, A. M., M. D., Professor of Anatomy and Surgery in the Medical Department of the University of the Pacific, San Francisco.

This is the first of a series of articles I design publishing on purpose to show truths which, for the most part, are in direct opposition to all established authority upon the subject, and are as follows:

1st. That atmosphere admitted into the joints or other tissues is not a source of irritation or injury, excepting where it acts mechanically; as when admitted into a vein by producing asphyxia, into the thoracic cavity by its pressure producing collapsion of the lungs, or when, by the long-continued exposure of a large amount of surface of any of the internal organs whose normal temperature is much above that of the atmosphere, it reduces it so as to produce a morbid action.

2d. That the division of entire ligaments about the joints is not only no impediment to their ultimate strength but facilitate the cure by enabling the surgeon to open the affected part fully, for the purpose of applying medical substances to the articular surfaces when these are ulcerated or otherwise diseased.

3d. That the only true mode of treating ulcerations of bone, however slight, within a joint, is to lay it open freely and apply remedial agents directly to the part affected.

4th. That opening the joints early in cases of matter burrowing in them is far more imperiously demanded than the opening of other parts thus affected, and the operation produces no further pain or inconvenience to the patient, in any respect, than when performed upon parts remote from the joints.

5th. That after opening a large joint, the knee, for instance, by an incision several inches long, the wound should be kept open by the introduction of lint or other similar substance until the parts within the articulation become healthy, and in all cases it should be made to heal by granulation.

6th. That extensive wounds opening freely the large joints, such as the knee, (even when lacerated as by a saw, which must necessarily heal by granulation,) do not as often give rise to violent symptoms as very small wounds, such as are made by the corner of a hatchet, an adze, or a penknife, which heal on the outside by first intention.

7th. That there are no known limits beyond which a tendon or ligament will not be reproduced after division, provided the parts are made to heal by granulation, and that the present acknowledged rule of two inches being the maximum distance in which the divided ends of a ligament or tendon can safely be separated, has not the least foundation in fact. Each of the above propositions has been fully tested by experience in numerous cases, which, during the course of this series of articles, shall be drawn upon as largely as brevity will admit.

CASE 1st.—Mr. A. J., æt. 29, received a penknife wound in the knee-joint, immediately on the outer side of the patella, which being small and causing little inconvenience, gave him no concern whatever.

The wound healed by first intention on the surface, and he continued his work as drayman as usual for two weeks, having not the least suspicion that mischief was brewing.

At the end of that time, however, the joint began to inflame, and shortly after attended with the most excruciating pain. The inflammatory action rapidly extending, the tissues of the whole joint were soon involved, and in a week more, when I was called, extensive fluctuation could be distinctly felt not only about the articulation, but in the lower part of the thigh. Chloroform and morphine had been used extensively, affording only temporary relief from the intense pain.

The case being a common one, I at once opened the joint freely by two incisions, eight inches long each, just back of the patella, on the internal and external side of the leg, which gave exit to nearly a quart of purulent matter, which was burrowing in the joint and lower part of the thigh. The smarting of the incisions had hardly subsided before the patient pronounced himself relieved, and the following night slept as well as if nothing had been the matter. The incisions were filled with lint, wet in an evaporating lotion composed of one part of alcohol and ten of water; a roller wet in the same was applied all over the limb as tightly as the patient could conveniently bear, commencing at the foot. About twenty-five ounces of spr. mindereri were given every twenty-four hours for the first three days, and an opiate administered occasionally.

On the fifth day, the wound being in a state of suppuration, the cold lotion was discontinued and poultices applied instead.

The roller was still continued upon the limb from the foot to the upper third of the thigh, a small opening simply being left at the most dependent portion of each incision. The poultices were applied outside of the roller.

The lint was permitted to remain in the wound for about two weeks, when it was removed. Tincture of iodine was applied every day all over the joint after suspending the use of the evaporating lotion.

A gentle motion was instituted about the tenth day, and kept up through the major part of convalescence, which lasted about nine weeks, when the patient was able to walk comparatively well. He improved rapidly after that until recovery was complete, though the wound was not entirely cicatrized for over five months. Not the least immobility followed in this case, and the patient recovered completely in every respect.

*Remarks.*—In this case a single incision would doubtless have answered the purpose, though not so well as two. The true plan of operation in these cases is not only to discharge every drop of purulent matter that may be collected, but likewise prevent any more collecting; and free incisions kept well open until the parts inside become healthy, together with a roller tightly applied to the limb, are the means of securing this object. The operation is not a severe one when well performed, as it may be done safely with great rapidity.

The knee-joint is surrounded by a large number of tendons covered with sheaths lined by bursæ mucosa, which on being wounded are liable to cause the burrowing of purulent matter among the surrounding parts, and may thereby give rise to symptoms almost as violent as when the matter forms in the joint itself; and though not so apt to generate a disorganizing disease of the joint, still, if neglected, this often would occur, and it is difficult to ascertain before an operation whether matter has formed internal or external to the capsular ligament. In the treatment, however, it makes but little difference whether the capsular ligament contains the pus or not, so far as the operative procedure is concerned, because it is nearly the same in both cases.

The surgeon should be sure that he opens the parts to a sufficient extent to admit of the discharge of all the purulent matter that may be accumulated, and it is immaterial whether he involes the joint not or in the operation. It is necessary to keep the incisions well open, otherwise matter might burrow still after the operation, and the worst consequences ensue.

To sum up, it is the accumulation of purulent matter that is to be prevented or removed in the treatment of injuries about the joints; and without this, all remedial measures will be abortive, and local, and constitutional symptoms of the highest grade will come on, jeopardizing the limb, if not the life of the patient.

When matter forms between the deep-seated fascia and capsular ligament, involving the bursæ mucosa lining the sheaths of tendons about the knee, the pain is almost as great, as when within the capsular ligament.

The bursæ mucosa being the same in structure as mucous membranes, are disposed to suppurate under slight inflammation; and being extensive here, pus is rapidly formed as soon as the parts are lighted up by inflammation.

Case 2d.—M. R. æt. 24, received a wound on the outer side of the knee by the corner of a sharp new hatchet, which gave exit to a drop or two of blood. The external wound was but about half an inch in length, and, as it gave him no pain, was not the source of the least anxiety, and the patient continued his employment of day laborer as usual for a week. At the end of that time the knee became painful, which induced him to go to bed. From this time on, for five days, when I was called, the pain he suffered was most agonizing. Finding fluctuation all over the knee, I at once made an incision seven inches long, which gave exit to more than a pint of purulent matter, and with it perfect relief. After the pus had been discharged it was found that the capsular ligament had not been opened, but that the pus had collected between it and the deep-seated fascia, which had been freely opened by the knife.

After Treatment.—The after-treatment was the same as in case first, excepting that the tincture of iodine was not used. Gentle motion was instituted, in about one week from the time of the operation, and continued more or less every day, until the patient recovered sufficiently to walk, which was seven weeks. He has since recovered perfectly, without the least weakness or immobility of the joint.

Remark.—The incision was made on the outer side of the knee, which is the point of election in all cases where one incision only is made, for the better discharge of purulent matter in or about this joint, seeing that the patient nearly always wishes to take a position on his back, with the knees separated, and the diseased limb flexed, which brings the wound on the outside of the knee, in the most dependent position. Without giving this matter due consideration, I have occasionally operated differently, but seldom with entirely satisfactory results. In the next two articles I shall give cases of division and reproduction of the ligamentum patellæ.—*American Medical Gazette.*

